# ANXIETY AND DEPRESSIVE SYMPTOMS MEDIATE THE LINK BETWEEN PERCEIVED NEIGHBORHOOD CHARACTERISTICS AND COGNITION

**DOI:** 10.1093/geroni/igz038.248

**Published:** 2019-11-08

**Authors:** Neika Sharifian, Neika Sharifian, Afsara B Zaheed, Briana N Spivey, Laura B Zahodne

**Affiliations:** 1 University of Michigan, Ann Arbor, Michigan, United States; 2 Spelman College, Lithonia, Georgia, United States

## Abstract

Although prior research has linked perceived neighborhood characteristics to cognition, scant research has investigated underlying mechanisms regarding how neighborhood characteristics impact cognition. One pathway, in particular, may be through mental health outcomes. Poorer neighborhood characteristics have been independently linked to greater depressive and anxiety symptoms, which may, in turn, be risk factors for cognitive decline in later life. The current study examined direct and indirect effects of perceived neighborhood characteristics (social cohesion, physical disorder) on cognitive functioning (episodic memory, executive functioning) through anxiety and depressive symptoms using longitudinal data from the Health and Retirement Study (2010–2014). Results revealed that higher social cohesion was associated with better memory and executive functioning through lower anxiety and depressive symptoms. Physical disorder was associated with worse episodic memory and executive functioning through greater anxiety symptoms. These findings highlight the importance of neighborhood context for promoting both mental and cognitive health outcomes in older adulthood.

